# Methods for bone quality assessment in human bone tissue: a systematic review

**DOI:** 10.1186/s13018-022-03041-4

**Published:** 2022-03-21

**Authors:** Fangxing Wang, Leyu Zheng, Jan Theopold, Stefan Schleifenbaum, Christoph-Eckhard Heyde, Georg Osterhoff

**Affiliations:** 1 ZESBO - Center for Research On Musculoskeletal Systems, Department of Orthopedic Surgery, Traumatology and Plastic Surgery, Leipzig University, Semmelweisstraße 14, 04103 Leipzig, Germany; 2 Department of Orthopedic Surgery, Traumatology and Plastic Surgery, Leipzig University, Liebigstraße 20 Haus 4, 04103 Leipzig, Germany

**Keywords:** Bone quality, Imaging, Mechanical testing, Bone composition

## Abstract

**Background:**

For biomechanical investigations on bone or bone implants, bone quality represents an important potential bias. Several techniques for assessing bone quality have been described in the literature. This study aims to systematically summarize the methods currently available for assessing bone quality in human bone tissue, and to discuss the advantages and limitations of these techniques.

**Methods:**

A systematic review of the literature was carried out by searching the PubMed and Web of Science databases from January 2000 to April 2021. References will be screened and evaluated for eligibility by two independent reviewers as per PRISMA (Preferred Reporting Items for Systematic Reviews and Meta-Analyses) guidelines. Studies must apply to bone quality assessment with imaging techniques, mechanical testing modalities, and compositional characterization. The terms used for the systematic search were: “(bone quality”. Ti,ab.) AND “(human bone specimens)”.

**Results:**

The systematic review identified 502 relevant articles in total. Sixty-eight articles met the inclusion criteria. Among them, forty-seven articles investigated several imaging modalities, including radiography, dual-energy X-ray absorptiometry (DEXA), CT-based techniques, and MRI-based methods. Nineteen articles dealt with mechanical testing approaches, including traditional testing modalities and novel indentation techniques. Nine articles reported the correlation between bone quality and compositional characterization, such as degree of bone mineralization (DBM) and organic composition. A total of 2898 human cadaveric bone specimens were included.

**Conclusions:**

Advanced techniques are playing an increasingly important role due to their multiple advantages, focusing on the assessment of bone morphology and microarchitecture. Non-invasive imaging modalities and mechanical testing techniques, as well as the assessment of bone composition, need to complement each other to provide comprehensive and ideal information on the bone quality of human bone specimens.

**Supplementary Information:**

The online version contains supplementary material available at 10.1186/s13018-022-03041-4.

## Introduction

As humans age, the rate of bone resorption by osteoclast cells outpaces the rate of bone formation. The mineral content of aged bones declines, eventually resulting in osteoporosis—a condition in which bones become more fragile and prone to fractures [1]. In accordance with World Health Organization (WHO) criteria, 10% of US women older than 50 years had osteoporosis and another 49% had osteopenia at the femur neck in 2005–2006 [2]. In 2010, osteoporosis affected roughly 22 million women and 5.5 million men in the European Union. In view of the variety of fragility fractures, including hip fractures, vertebral fractures, forearm fractures, the estimated economic burden is €37 billion per year [3].

Hence, research on osteoporotic fractures has increased over the past decades. Although bone mineral density (BMD) is considered to be the gold standard for the evaluation of bone strength and fracture risk [4], bone strength is determined by many other factors as bone microstructure, and bone components [4].

Besides methods for bone quality assessment that have been established in the clinical context, there are methods available to directly analyse the mechanical strength of bone tissue, such as micro-indentation, or nano-indentation tests [5, 6].

The aim of this study was to systematically summarize the current techniques commonly used to assess bone quality in human bone specimens, as well as the advantages and limitations of these methods.

## Methods

The PRISMA (Preferred Reporting Items for Systematic Review and Meta-Analyses) checklist and algorithm [7] was used to conduct a systematic review of the literature to find all studies concerning the bone quality assessment of human bone specimens. Since data collection has already been completed at the time of PROSPERO registration, this review could not be registered with PROSPERO.

No primary personal data were collected; therefore, no additional ethical approval needed to be obtained.

### Information source

A systematic review of the literature was searched by PubMed and Web of Science databases from January 2000 to April 2021. The language of the journal was limited to English, and the searched species was selected to be “human”. To further extend the search, the “similar articles” option of PubMed was employed in each paper.

### Search strategy

**Fig. 1:**
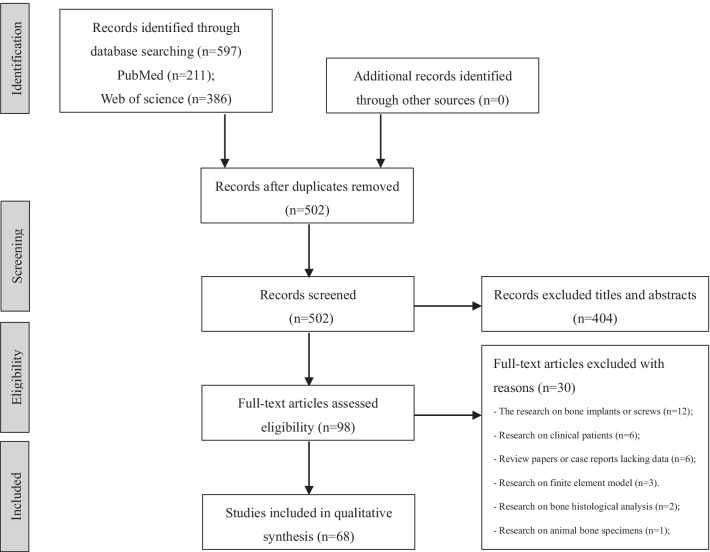
Preferred reporting item systematic reviews and meta-analysis (PRISMA) flow diagram of study selection

A search was performed independently by two reviews (F.W. and L.Z.), and terms were used for the systematic search: “(bone quality”. Ti,ab.) AND “(human bone specimens). After removing duplicates, the reviewers scanned the search results by titles and abstracts. After identifying potentially pertinent articles, full-text articles were sourced and checked for suitability according to the inclusion and exclusion criteria (Fig. 1). Any controversy between the two authors was sent and discussed with a third independent author.

### Eligibility criteria

Studies were selected on the basis of the following inclusion criteria: (a) in vitro experiments regarding the bone quality assessment of human bone specimens; (b) research on bone composition (DBM, organic composition); (c) articles published in English. Exclusion criteria were: (a) no access to full text; (b) case reports and review papers; (c) studies on bone implants or screws, bone histological analysis, and clinical patients; (d) non-English language publications; (e) studies on animal bone specimens; (f) studies on finite element analysis (FEA) models.

### Data extraction and analysis

Two authors (F.W. and L.Z.) independently performed data extraction and recorded this data using standard spreadsheet software (Excel for Mac 2016, version 16.2.9, Microsoft, Redmond, WA, USA). This included testing methods, authors and year of publication, journal of publication, study design, number of bone specimens, age, the site of specimens, main findings or summaries.

### Assessment of study quality

The Newcastle–Ottawa Scale (NOS) [8] which contains three primary components: selection, comparability, and exposure/outcome, is being used to evaluate the quality of non-randomized trials. For this review, the quality of all studies, including bias, was assessed using the adapted Newcastle–Ottawa Quality Assessment Scale (Additional file 1). According to the total quality score, studies were evaluated with the highest score of eleven, as unsatisfactory (0–5), satisfactory (6–8), and good (9–11), which refers to a published article [9]. Two authors (F.W., and L.Z.) assessed all the included articles independently. Disagreements were recorded by discussion.

### Statistical analysis

Continuous variables were described by the mean and standard deviation or median and range. Categorical variables were expressed with absolute and relative frequencies. Statistical significance is defined with *P* < 0.05. The large heterogeneity and lack of randomized controlled trials made it impossible to perform a meta-analysis. Furthermore, since the distributions of some indicators were only ranges, no other statistical analysis was possible.

## Results

### Study description and quality assessment

**Fig. 2:**
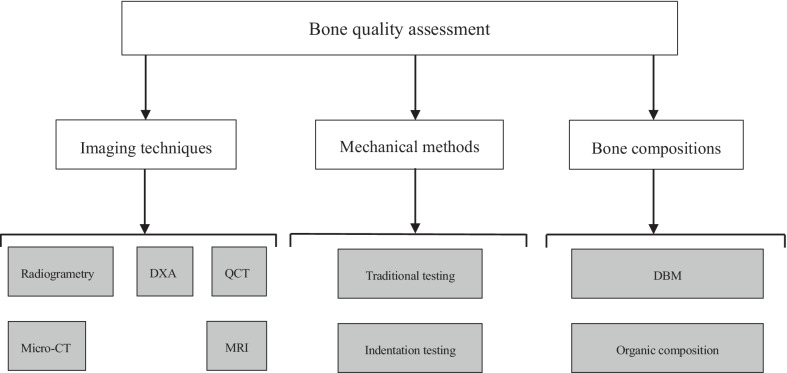
Different testing methods of bone quality, as well as the intrinsic bone composition that affects bone quality

**Fig. 3:**
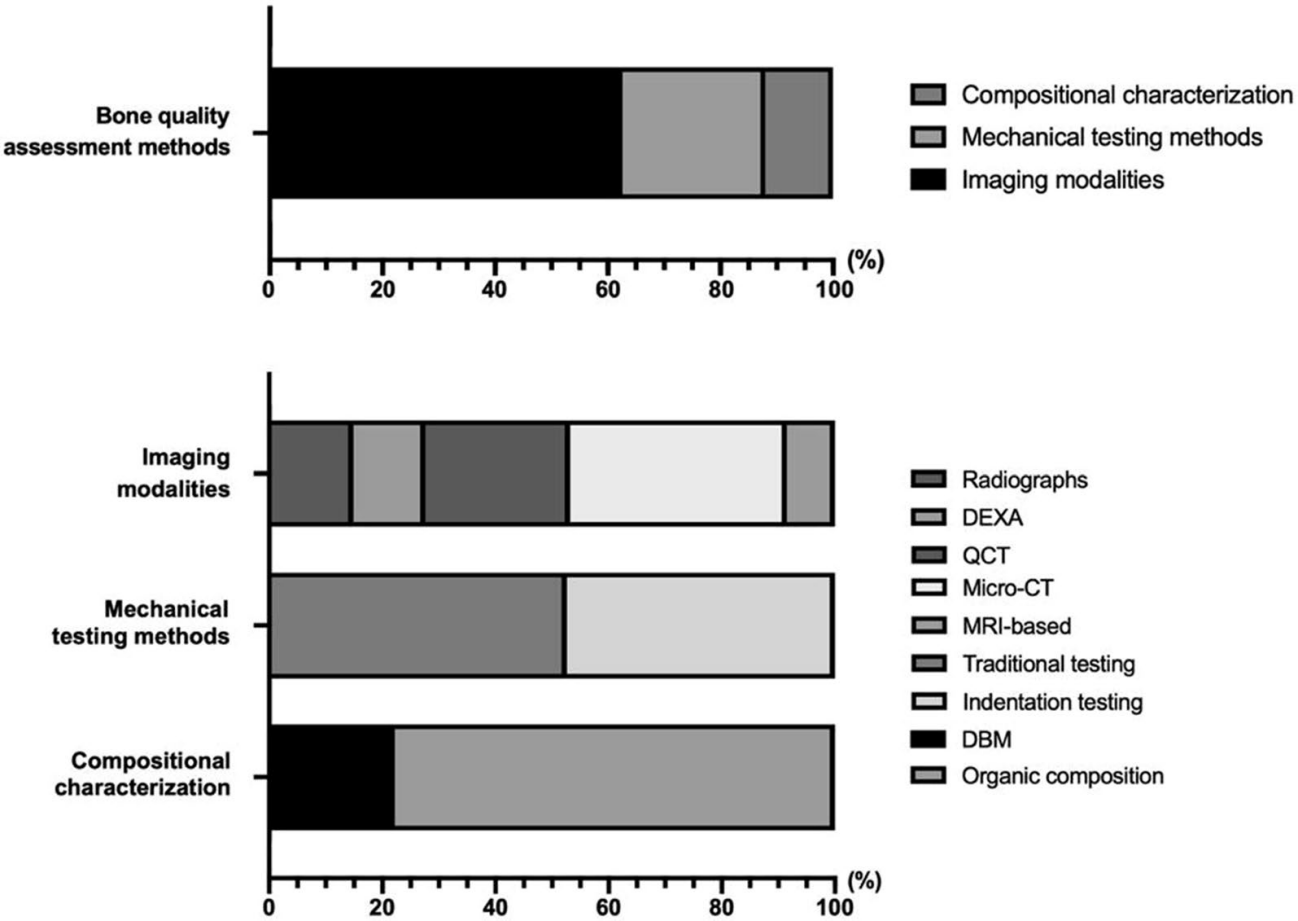
The percentage of different modalities in assessing bone quality

After scrutinizing the titles and abstracts, as well as examining the full texts, the remaining sixty-eight studies were included in the systematic review. Among them, forty-seven articles investigated several imaging modalities, including radiography, dual-energy X-ray absorptiometry (DEXA), CT-based techniques, and MRI-based modalities. Nineteen articles dealt with mechanical testing approaches, including traditional testing methods and novel indentation techniques. Nine articles reported the correlation between bone quality and bone composition, such as DBM, organic composition (Figs. 2 and 3).

A total of 2898 human cadaveric bone specimens were included (Table 1). The number of specimens ranged from 4 to 189 with a mean of 42.62 ± 42.39. Each bone quality assessment method has its advantages and limitations, the details see Table 2. The individual scores of each study are recorded in Table 3. Overall, 4, 52, and 12 of the included studies were rated as “unsatisfactory”, “satisfactory”, and “good”, respectively.

**Table 1:** Summary of methods of bone quality with important outcomes, advantages, and limitations

Testing methods	Authors and year of publication	Journal of publication	Study design	Number of specimens	Age (years)	The site of specimens	Main findings or summaries
Radiographs	Tingart, M. J. et al. 2003	The Journal of Bone and Joint Surgery	CTI	19	72 ± 11	Humeri	The cortical thickness of the proximal diaphysis is a reliable predictor of the bone quality of the proximal humerus
Radiographs	Ebraheim N. et al. 2000	Spine	Internal architecture	7	57–78	Sacrum	The strongest part of the sacrum is the anterior cortex above the foramina in S1 and S2. The weakest point of the sacrum was found to lie at the level of the junction of S2 and S3
Radiographs	Huber, M. B. et al. 2009	Medical Physics	BMD, texture information	14	70.8 (66.1–73.2)	Femoral specimens	Texture information contained in trabecular bone structure visualized on radiographs may predict whether an implant anchorage can be used and may determine the local bone quality from preoperative radiographs
Radiographs	Thevenot, J. et al. 2013	Journal of Bone and Mineral Research	BMD, THI	178	79.3 ± 10.4	Femoral bone	Conventional radiography is a low‐cost method for evaluating geometry, structure, and fracture risk of bone
Plain radiographs, DEXA, pQCT	Clavert, P. et al. 2016	Surgical Radiologic Anatomy	BMD, CMI	21	NR	Distal humerus	More than a direct evaluation of the bone density with a CT-scan, the cortio-medullar index (CMI) of the distal humerus diaphysis is a predictor of the bone quality of the distal humerus
DEXA	Tan, J. S. et al. 2010	The Spine Journal	BMD	189	NR	Lumbar specimens	In vitro BMD scan on explanted specimens measured lower DEXA values than in situ BMD scans on full cadavers. A correction factor when used resulted in more accurate measure of the in situ BMD
DEXA	Hua Y. et al. 2009	Clinical Oral Implants Research	Fractal analysis, morphometry	19	NR	Mandibular bone	They investigated the accuracy of fractal analysis and morphometry for bone quality assessment as measured with DEXA
DEXA	Choel, L. et al. 2003	Oral Surgery Oral Medicine Oral Pathology, and Oral Radiology	BMD, BMC	63	80.8 ± 10, 82.7 ± 7.3	Mandibular bone	The intra-alveolar trabecular bone of these 21 mandibles is affected by the same local and systemic influences as cortical bone, whereas the infra-alveolar trabecular bone is mostly sensitive to dental status
DEXA	Yang, J. et al. 2012	Journal of Biomechanical Engineering	BMD, BMC	9	NR	Femurs	The proposed technique is capable of detecting differences in bone quality. The ability to measure site-specific properties without exposure to radiation has the potential to be further developed for clinical applications
DEXA, QCT	Johannesdottir, F. et al. 2017	Bone	BMD, microstructures	76	74 ± 8.8	Proximal femurs	Both cortical and trabecular bone contribute to femoral strength, the contribution of cortical bone being higher in femurs with lower trabecular bone density
pQCT	Chaplais E et al. 2014	BMC Musculoskelet Disord	Material properties of bone	11	75 (59–93)	Leg	This protocol extends the capabilities of pQCT to evaluate bone quality in people who may be at an increased risk of metatarsal insufficiency fractures
HR-pQCT	Kirchhoff C. et al. 2012	BMC Musculoskelet Disord	General morphology	64	72.3 ± 17.4	Humeral head	The presented microarchitectural data measured by HR-pQCT allow for future subtle biomechanical testing comprising knowledge on age- and sex-related changes of the tuberosities of the humeral head
HR-pQCT	de Jong, J. J. et al. 2016	The Journal of Bone and Joint Surgery	Bone parameters	15	62–90	Distal radial	HR-pQCT can be used a promising tool to assess the fracture-healing process in patients with fiberglass cast
HR-pQCT; micro-CT	Liu X.S., Sekhon K.K. et al. 2010	J Bone and Mineral Research	Microstructural of human distal tibia	19	70.6 (55–84)	Tibia	Microstructural measurements and mechanical parameters of distal tibia can be efficiently derived from HR-pQCT images and provide additional information regarding bone fragility
HR-pQCT; micro-CT	Jorgenson, B. L. et al. 2015	Bone	Cortical porosity and density	23	66.3 (55–85)	Mid-shaft region of tibiae	The accuracy of the threshold-based method will improve as new HR-pQCT systems emerge and provide a robust quantitative approach to measure cortical porosity
pQCT	Diederichs, G. et al. 2006	Archives of Orthopaedic and Trauma Surgery	Regional BMD	88	75.8 ± 13.5	Humeri	Bone quality at the humeral head is best predicted by BMD measurements at the contralateral location rather than the ipsilateral distal site
HR-pQCT	Manske, S. L. et al. 2015	Bone	Bone microarchitecture	20	70 (49–95)	Radii	These data support the application of analysis techniques in HR-pQCT that are analogous to those traditionally used for micro-CT to assess trabecular microarchitecture
QCT	Mann, C. et al. 2018	Scientific Reports	BMD	10	80 (59–92)	Lumbar spine	A well-established alternative to DXA is QCT, a three-dimensional method which measures trabecular BMD in milligrams per cubic centimeter by indirectly quantifying hydroxyapatite in comparison with a reference phantom
QCT	Zheng Y et al. 2000	Spine	BMD	13	31 (24–36)	Sacrum	This report detailed BMD variations of the S1 body and ala in a young male group of specimens
pQCT	Lu WW. et al. 2000	Clinical Orthopaedics and Related Research	BMD	13	31 (24–36)	Sacrum	The highest bone mineral density in the lumbosacral spine is found at the pedicles and regions closest to pedicle bases
Micro-CT	Lee, J. H. et al. 2017	Journal of Periodontal &Implant Science	3D-microstructure	60	75.7 (67.3–96)	Hemimaxillae	Bone quality depended on trabecular separation (Tb.Sp) and number—that is, endosteal space architecture—rather than bone surface and trabecular thickness (Tb.Th). Regardless of bone quality, Tb.Th showed little variation
Micro-CT	Chen, R. E. et al. 2019	Clinical Orthopaedics and Related Research	BMD, CTI	10	63 (59–67)	Distal clavicular regions	In the distal clavicle, BMD and cortical thickness are greatest in the conoid tubercle and intertubercle space
Micro-CT	Xie, F. et al. 2018	Archives of Osteoporosis	Microstructural properties	67	NR	Spinous processes	Post-menopausal women and older men with osteoporosis have worse bone quality in autografts than non-osteoporotic men and women. Postmenopausal women with osteoporosis presented serious microarchitectural deterioration in older population
Micro-CT	Ding, M. et al. 2012	Bone	microarchitectural, mechanical, collagen and mineral properties of normal adolescent cancellous bone	23	NR	Left proximal tibiae	Micro-CT can be used to measure various parameters, such as 3D microarchitecture, mechanical properties, collagen and mineral properties of adolescent cancellous bone
Micro-CT, radiography	Rupprecht, M. et al. 2006	Journal of Orthopaedic Research	Bone microarchitecture	60	NR	Calcanei	Bone mass and structure are risk factors in respect to the occurrence and severity of calcaneal fractures, and indicate that calcaneal fractures are at least in part osteoporotic fractures
Micro-CT	Greenwood, C. et al. 2018	Aging and Disease	Bone microarchitecture	164	21–93	Femoral heads	Micro-computed tomography was utilised to investigate the microarchitecture of femoral head trabecular bone from a relatively large cohort of non-fracture and fracture human donors
Micro-CT	Kuhn, G. et al. 2007	Journal Homo of Comparative Human Biology	Bone surface structures, microarchitecture	5	NR	Postcranial	Micro-CT is a tool of high value for the examination of postcranial bone disorders. It cannot replace histological examinations completely because it cannot assess the bone quality (woven or lamellar)
Micro-CT	Arnold, E. L. et al. 2020	Journal of the mechanical behaviour of biomechanical materials	BMD, TMD, microarchitectural parameters	100	20–93	Femoral heads	Properties which are not age dependent are significantly different between age-matched non-fracture and fracture specimens, indicating osteoporosis is a disease, and not just an accelerated aging process
Micro-CT	Ding, M. et al. 2003	The Journal of Bone and Joint Surgery	3D microstructural properties	120	73 (63–81); 72 (58–85)	Proximal tibiae	Using unbiased 3-D methods, we have demonstrated microstructural changes in subchondral cancellous bone in human tibial early OA
Micro-CT	Marinozzi, F. et al. 2012	Ann Ist Super Sanita	3D-structure, morphometric parameters	6	NR	femoral heads	Micro-CT is a promising technique for trabecular bone analysis. Bone morphometric parameters obtained by microtomographic processing allows to completely characterize human bone
Micro-CT	Kim, Y. J. et al. 2015	Clinical Implant Density and Related Research	BMD, 3D-microarchitecture	34	NR	Jaw	Two aspects of bone density using micro-CT, the BV/TV and BMD, are highly correlated with 3D micro-architecture parameters, which represent the quality of trabecular bone. This noninvasive method may adequately enhance evaluation of the alveolar bone
Micro-CT	Kamal, M. et al. 2018	The Journal of Craniofacial Surgery	BMD, structural morphometric	60	69.5 (57.3–81.2)	Calvarium, maxillary tuberosity, mandibular ramus, mandibular symphysis, anterior iliac crest, and tibia	The results show great variation in bone densities and 3D morphometric values across different donor sites
Micro-CT	Thomsen J.S. et al. 2013	Bone	BV/TV, Tb.Th, Tb.N, SMI, CD, DA	79	21.7–96.4; 22.6–94.6	Second lumbar vertebral (L2)	Vertical and horizontal oriented bone density decreases with age in both women and men, and that vertical oriented bone is lost more quickly in women than in men,
NMR	Ni, Q.W. et al. 2007	Measurement Science and Technology	Bound and mobile water	10	65.9 (51–87)	Femurs	Bound to mobile water may be used as a measure of bone quality describing both porosity and water content, both of which may be important determinants of bone strength and fracture resistance
HR-MRI	Link, T. M. et al. 2003	European Radiology	Trabecular bone structure	39	76.9 ± 7.2	Distal radius	High-resolution MR-derived structure parameters, however, performed better in the prediction of trabecular bone structure
MRI (HR-MRI)	Vieth V. et al. 2001	Investigative Radiology	Trabecular bone structure parameters	30	68.5 ± 8.2	Calcaneus	Trabecular bone structure depicted by HR-MRI is significantly correlated with that shown in macro-sections
Micro-MRI	Liu, X. S., Rajapakse C. S. et al. 2010	Journal of Bone and Mineral Research	3D model-independent microstructural measurements	25	70.6 (55–84)	Distal tibias	Most microstructural and mechanical properties of the distal tibia can be derived efficiently from micro-MR images and can provide additional information regarding bone quality
Cyclic compressive loading	Goff, M. G.et al. 2015	Bone	Bone microdamage	32	78 ± 8.8	Vertebral cancellous bone	Microdamage accumulation in fatigue is likely dominated by heterogeneity in tissue material properties rather than stress concentrations caused by micro-scale geometry
Compression-tension loading	Bevill, G. et al. 2006	Bone	Bone volume fraction and architecture, bone strength	54	70 ± 11	Femoral neck, greater trochanter, proximal tibia, vertebral body	Within very low-density bone, the potentially important biomechanical effect of large-deformation failure mechanisms on trabecular bone strength is highly heterogeneous and is not well explained by standard architectural metrics
Compression test	Ding M et al. 2001	Acta Orthop Scand	Mechanical and compositional properties	10	73 (63–81)	Proximal tibiae	Cancellous bone quality is reflected by the amount of bone tissue present, the mechanical properties of the tissue, and its trabecular architecture
Compression test	Kalouche, I. et al. 2010	Clinical biomechanics	Mechanical properties	82	88.9 (76–96)	Cadaveric shoulders	Good correlation between apparent density and elastic modulus was found only in the sagittal planes but not in the coronal and axial plane
Compression test	Bayraktar, H. H. et al. 2004	Journal of Biomechanics	Elastic and yield properties	94	65.5 ± 9.1; 71.8 ± 8.8	Femoral neck	The elastic modulus and yield strains for trabecular tissue are just slightly lower than those of cortical tissue, because of the cumulative effect of these differences, tissue strength is about 25% greater for cortical bone
Micro-indentation	Dall'Ara, E. et al. 2012	Bone	Bone microdamage	35	44–82	Thoracolumbar vertebral bodies (T12-L5)	Micro-indentation was found to discriminate between highly damaged and intact tissue in both trabecular and cortical bone tested in vitro. It remains to be investigated whether this technique would be able to detect also the damage
RPI, bending test	Granke, M. et al. 2014	Journal of the mechanical behaviour of biomechanical materials	Tissue anisotropy, mechanical behaviour	26	25–101	Femoral mid-shaft	With a transverse isotropic behaviour akin to tissue hardness and modulus as determined by micro- and nanoindentation and a significant association with toughness, RPI properties are likely influenced by both elastic and plastic behaviour of bone tissue
RPI	Jenkins, T. et al. 2015	Journal of the mechanical behaviour of biomechanical materials	Maximum load, sample orientation, mode of use, sample preparation and measurement spacing	5	67–89	Femoral heads	RPI users can minimize the potential confounding effects associated with the variables investigated here and reduce the coefficient of variation, hence achieving more consistent testing
Nanoindentation	Albert, C. et al. 2013	Clinical biomechanics	Bone tissue elastic modulus and hardness	11	5–18	Lower extremity long bones	Nanoindentation can be used to measure bone material properties, providing valuable data
Cyclic fatigue loading; Micro-CT	Lambers, F. M.et al. 2013	PLoS One	Mechanical properties, microdamage and bone microarchitecture	32	76 ± 8.8	The third lumbar vertebral bodies	Even small amounts of microscopic tissue damage in human vertebral cancellous bone may have large effects on subsequent biomechanical performance
Micro-CT, compression testing	Charlebois, M. et al. 2010	Journal of Biomechanics Engineering	Volume fraction, compressive behaviour	148	53–100	T12 vertebrae, distal radii, femoral head, calcanei	Reasonable predictions of their compressive mechanical behaviour can be made using the volume fraction and fabric over a broad range of strains
Radiographs, Micro-CT, compressive loading	Yeni, Y. N., Wu B., et al. 2013	Journal of Biomechanics Engineering	Microstructure at various levels of compressive deformation	7	NR	Femoral and tibial cancellous bone cylinders	The heterogeneity of the microstructure is especially sensitive to deformation and these could be good parameters to use to estimate strain history in the tissue
QCT, uniaxial compression test	Wachter NJ. et al. 2001	Clinical Biomechanics	Singh index, mechanical competence	31	68.3 ± 11.7	Femurs	Assessment of bone mineral density by QCT is a reliable and precise method for the estimation of cancellous bone material properties
NMR, three-point bending testing	Nyman, J S. et al. 2008	Bone	Mobile and bound water; Bone strength and toughness	18	66.3 (47–87)	Femurs	Quantifying mobile and bound water with magnetic resonance techniques could potentially serve as indicators of bone quality
Bending test, CT scanner	Lettry, S. et al. 2003	Bone	Mechanical properties, CT numbers	5	85.8 (53–106)	Mandible	A weak correlation was found between the modulus values and the CT number of the mandible. This would not be sufficient for accurate predictions of the bone properties from CT scans
Micro-CT, compression test	Karim, L. et al. 2011	Journal of Orthopaedic Research	Bone microdamage	26	18–97	Tibial plateaus	Low bone volume fraction and increased structure model index have strong influences on microdamage accumulation in bone through altered initiation
Micro-CT, micro-indentation, and bending test	Merlo, K. et al. 2020	Journal of Orthopaedic Research	Microarchitecture, Mechanical Properties, and AGEs	40	73.1 ± 10.9	Tibias	The accumulation of AGEs would cause lower elastic modulus and lower fracture toughness in human cortical bone
Micro-CT, RPI	Beutel, B. G. et al. 2015	Bone	BV/TV, Porosity, Mechanical outputs	6	79 (76–88)	Tibiae	RPI parameters will help to further facilitate its use as a clinical diagnostic tool
RPI, bending test	Krege J.B. et al. 2016	Bone	IDI, TID, bone toughness	4	76–85	Femora	RPI measurements alone, as compared to bending tests, are insufficient to reach conclusions regarding mechanical properties of bone
Indentation testing, CT scanner	Zumstein, V. et al. 2012	Journal of Shoulder and Elbow Surgery	Mechanical strength, subchondral mineralization	32	80.5 (59–95)	Shoulder	Mechanical strength and subchondral mineralization in the humeral head are significantly associated
X-ray radiograms, tensile fracture toughness	Yeni, Y. N. Brown C. U.et al. 2013	Journal of the mechanical behaviour of biomechanical materials	Femoral cortex geometry, tissue mechanical properties	25	53.3 ± 19.7	femurs	Fracture toughness of the tissue was significantly related to radiogram metric indices and that some of these indices explained a greater variability in toughness than porosity, age or gender
Micro-CT, nanoindentation, compressive loading	Li, Z. C. et al. 2012	Arthritis Rheum	Fatigue strength, microarchitecture, mineralization degree, and biomechanical properties	60	53–86; 59–87	Femoral head	The difference in mechanical properties between osteoarthritis and osteoporosis cancellous bone is attributed to different bone mass and bone structure
Compressive loading, microscopic analysis	Hernandez, C. J. et al. 2014	Bone	Mechanical properties, BV/TV and microdamage	47	64–92	Vertebral cancellous bone	Small amounts of microdamage do not necessarily indicate impaired mechanical performance, the presence of modest amounts of microdamage is always indicative of large reductions in cancellous bone stiffness and strength
Bending test, RPI, nanoindentation	Katsamenis, O. L. et al. 2015	Bone	Fracture toughness, crack growth resistance	4	63.25 (43–83)	Femurs	RPI is an emerging technique with the clinical potential for the direct assessment of the mechanical properties of the bone
Bone composition; Compression test	Follet, H. et al. 2004	Bone	DBM, mechanical properties	20	78 ± 8	Calcaneus	The increase in bone strength when DBM is modified in a physiological range without necessary changes of bone matrix volume and bone microarchitecture
Bone composition	Saito, M. et al. 2006	Calcified Tissue International	DBM, collagen crosslinking	50	78 ± 6 77 ± 6	Hip	Detrimental crosslinking in both low and high mineralized bone result in impaired bone quality in osteoporotic patients
Bone composition	Karim, L. et al. 2012	PLoS One	Heterogeneous glycation	42	59.3 ± 22.1	tibial plateaus	The extent of NEG in tibial cancellous bone was the dominant predictor of bone fragility and was associated with changes in microarchitecture and microdamage
Bone composition	Willett, T. L.et al. 2019	Bone	bone collagen integrity parameters, fracture toughness	54	64.4 ± 21.3	Femurs or femur mid-shafts	Bone collagen integrity as measured by thermomechanical methods is a key factor in cortical bone fracture toughness
Bone composition	Poundarik, A. A. et al. 2015	Journal of the mechanical behaviour of biomechanical materials	Glycated collagen	9	34–85	Tibiae	Advanced glycation end-products (AGEs) are predictive of bone quality in aging humans and have diagnostic applications in fracture risk
Bone composition	Ural, A. et al. 2015	Osteoporosis international	NEG	96	60.6 ± 21.0	Proximal end of tibiae	AGEs alter the resorption process and/or accumulate in the tissue as a result of reduced resorption and may lead to bone fragility by adversely affecting fracture resistance through altered bone matrix properties
Bone composition	Wang X et al. 2002	Bone	Collagen molecular structures, mechanical integrity of the collagen network, mechanical properties of bone	30	19–89	Femurs	The adverse changes in the collagen network occur as people age and such changes may lead to the decreased toughness of bone. Also, the results suggest that nonenzymatic glycation may be an important contributing factor causing changes in collagen and, consequently, leading to the age-related deterioration of bone quality

CTI: Cortical thickness; 2D: Two-dimensional; 3D: Three-dimensional; DEXA: Dual-energy X-ray absorptiometry; BMD: Bone mineral density; BMC: Bone mineral content; Micro-CT: Micro-computed tomography; μMRI: Micro-magnetic resonance imaging; BV/TV: Bone volume fraction; Tb.Th: Trabecular thickness; Tb.Sp: Trabecular spacing; Tb.N: Trabecular number; BS/TV: Bone surface density; SMI: Structure model index; Conn.D.: Connectivity density; CD: Connectivity density; DA: Degree of anisotropy; HR-MRI: High-resolution magnetic resonance imaging; RPI: Reference point indentation; HR-pQCT: High-resolution peripheral quantitative computed tomography; CMI: Cortical-medullar index; THI: Trabecular homogeneity index; NMR: Nuclear magnetic resonance; IDI: Indentation distance increase; TID: Total indentation distance; DBM: Degree of bone mineralization; AGEs: Advanced glycation end products; NEG: Non-enzymatic glycation; TMD: Tissue mineral density

**Table 2:** Summary of studies characteristics, patient or specimen demographic details and main findings or summaries

	Category	Methods	Main indicators	Advantages	Limitations
Imaging modalities	X-ray-based modalities	Radiography	CTI, CMI, THI	Simplicity, low-cost, low radiation dose	Insufficient precision, 2D imaging
		DEXA	BMC, BMD	Low radiation dose, accuracy, simplicity	2D imaging, cannot capture the 3D micro-architecture
	CT-based modalities	QCT, pQCT, HR-pQCT	3D-morphology, BMC, BMD	High spatial resolution,, reproducible, 3D imaging, non-invasiveness	Larger radiation does, expensive equipment
		Micro-CT	3D-microstructure, BV/TV, Tb.Th, Tb.Sp; Tb.N, BS/TV, BS/BV, SMI, Conn.D	Comprehensive, high spatial resolution, 3D bone structure, non-invasiveness	Larger radiation does, expensive equipment
	MR-based modalities	NMR, HR-MRI, μMRI	3D bone geometry, trabecular morphology	High accuracy, no radiation, high-resolution 3D imaging, non-invasiveness	Expensive equipment, professional operation, more susceptible to image post-processing
Mechanical testing	Traditional testing	Compression, tension, bending, and torsion tests	Elastic modulus, Ultimate strength, Yield strength	Directness, accuracy, simplicity	Destructive testing, cannot be repeated
	Indentation testing	Macro-indentation, RPI, nano-indentation	Hardness, Brittleness	Directness, simplicity, minimally invasiveness	Its outcomes are relatively sole, limited to superficial sites, reliability and significance of parameters need to be validated further
Bone composition	–	Computerized quantitative contact microradiography method, HPLC, et al	DBM, Organic phases	An intrinsic effect on bone stiffness and strength	Not comprehensive enough

**Table 3:** Quality Assessment of the Studies by the Newcastle–Ottawa Scale

Study	Selection comparability outcome										Total (11/11)
	Selection			Comparability	Outcome						
	Representativeness of anatomical sites or factors	Representativeness of parametric data	Sample size	Comparability of test/controls on the basis of the analysis	Assessment of outcome	Assessment method	Outcome description	Specimen information (age)	Amount of specimens large enough	Statistical test	
Wang X. et al. 2002	★	★	0	★	0	★	★	★	0	★	7/11
Tang JS et al. 2010	★	★	0	★	0	★	★	0	★	★	7/11
Ebraheim N et al. 2000	★	0	0	★	0	★	★	★	0	★	6/11
Tingart MJ et al. 2003	★	★	★	★	0	★	★	★	0	★	8/11
Link TM et al. 2003	★	★	0	★	0	★	★	★	0	★	7/11
Kim YJ et al. 2015	★	★	0	★	0	★	★	0	0	★	6/11
Xie F et al. 2018	★	★	0	★	0	★	★	0	★	★	7/11
Chen RE et al. 2019	★	★	0	★	0	★	0	★	0	★	6/11
Greenwood C et al. 2018	★	★	0	★	0	★	★	★	★	★	8/11
Hua Y. et al. 2019	★	0	0	★	★	★	★	0	0	★	6/11
Choel L et al. 2003	★	★	0	★	0	★	★	★	★	★	8/11
Huber, M. B. et al. 2009	★	★	0	★	0	★	★	★	0	★	7/11
Johannesdottir, F. et al. 2017	★	★	0	★★	0	★	★	★	★	★	9/11
Yang J et al. 2012	★	★	0	★	0	★	★	0	0	★	6/11
Thevenot, J. et al. 2013	★	★	0	★	0	★	★	★	★	★	8/11
Kuhn, G. et al. 2007	★	★	0	★	0	★	0	0	0	0	4/11
Arnold, E. L. et al. 2020	★	★	0	★	0	★	★	★	★	★	8/11
Lee JH et al. 2017	★	★	0	★	0	★	★	★	★	★	8/11
Kamal M et al. 2018	★	★	0	★	0	★	0	★	★	★	7/11
Lu WW et al. 2000	★	0	0	★	0	★	0	★	0	★	5/11
Zheng Y et al. 2000	★	★	0	★	0	0	0	★	0	★	5/11
Mann, C. et al. 2018	★	★	0	★	0	★	★	★	0	★	7/11
Manske, S. L. et al. 2015	★	★	0	★	0	★	★	★	0	★	7/11
Diederichs, G. et al. 2006	★	★	0	★	0	★	★	★	★	★	8/11
Kirchhoff C. et al. 2012	★	★	0	★	0	★	★	★	★	★	8/11
Rupprecht, M. et al. 2006	★	★	0	★★	0	★	★	0	0	★	7/11
Albert, C. et al. 2013	★	★	0	★	0	★	★	★	0	★	7/11
Ding, M. et al. 2012	★	★	0	★	0	★	★	0	0	★	6/11
Jenkins, T. et al. 2015	★	★	0	★	0	★	★	★	0	★	7/11
Clavert, P. et al. 2016	★	★	0	★★	0	★	★	0	0	★	7/11
Katsamenis, O. L. et al. 2015	★	★	0	★★	0	★	★	★	0	★	8/11
Hernandez, C. J. et al. 2014	★	★	★	★★	0	★	★	★	0	★	9/11
Liu, X. S. et al. 2010	★	★	0	★	0	★	★	★	0	★	7/11
de Jong, J. J. et al. 2016	★	★	0	★★	0	★	★	★	0	★	8/11
Chaplais E et al. 2014	★	0	0	★★	0	★	0	★	0	★	6/11
Li, Z. C. et al. 2012	★	★	0	★★	0	★	★	★	★	★	9/11
Yeni, Y. N. et al. 2013	★	★	0	★★	0	★	★	0	0	★	7/10
Zumstein, V. et al. 2012	★	★	★	★★	0	★	★	★	0	★	9/11
Krege, J.B. et al. 2016	★	★	0	★★	0	★	★	★	0	★	8/11
Beutel, B. G. et al. 2015	★	★	0	★★	0	★	★	★	0	★	8/11
Merlo, K. et al. 2020	★	★	★	★★	0	★	★	★	0	★	9/11
Karim, L. et al. 2011	★	★	0	★★	0	★	★	★	0	★	8/11
Ni, Q.W. et al. 2007	★	★	0	★	0	★	★	★	0	0	6/11
Bayraktar, H. H. et al. 2004	★	★	0	★	0	★	0	★	★	★	7/11
Lettry, S. et al. 2003	★	0	0	★★	0	★	★	★	0	★	7/11
Lambers, F. M.et al. 2013	★	★	0	★★	0	★	★	★	0	★	8/11
Kalouche, I. et al. 2010	★	★	★	★	0	★	★	★	★	★	9/11
Ding M et al. 2001	★	★	0	★	0	★	★	★	0	★	7/11
Bevill, G. et al. 2006	★	★	0	★	0	★	★	★	★	★	8/11
Goff, M. G.et al. 2015	★	★	0	★	0	★	★	★	0	★	7/11
Dall'Ara, E. et al. 2012	★	★	0	★	0	★	★	★	0	★	7/11
Granke, M. et al 2014	★	★	0	★★	0	★	★	★	0	★	8/11
Follet, H. et al. 2004	★	★	0	★★	0	★	★	★	0	★	8/11
Ural, A. et al. 2015	★	★	0	★	0	★	★	★	★	★	8/11
Poundarik, A. A. et al. 2015	★	★	0	★	0	★	★	★	0	0	6/11
Nyman JS. et al. 2006	★	★	0	★	0	★	★	★	0	★	7/11
Willett, T. L.et al. 2019	★	★	0	★	0	★	★	★	★	★	8/11
Karim, L. et al. 2012	★	★	0	★	0	★	★	★	★	★	8/11
Saito, M. et al. 2006	★	★	0	★	0	★	★	★	★	★	8/11
Yeni, Y. N. et al. 2013	★	★	0	★★	0	★	★	★	0	★	8/11
Charlebois, M. et al. 2010	★	★	0	★★	0	★	★	★	★	★	9/11
Thomsen, J S. et al. 2013	★	★	0	★★	0	★	★	★	★	★	9/11
Wachter NJ. et al. 2001	★	★	0	★★	★	★	★	★	0	★	9/11
Jorgenson, B. L. et al. 2015	★	★	0	★★	★	★	★	★	0	★	9/11
Marinozzi, F. et al. 2012	★	★	0	★	0	★	0	0	0	0	4/11
Ding, M. et al 2003	★	★	0	★	★	★	★	★	★	★	9/11
Liu XS. et al 2010	★	★	0	★★	★	★	★	★	0	★	9/11
Vieth V. et al 2001	★	★	0	★	★	★	★	★	0	★	8/11

#### Imaging modalities

Imaging modalities for assessing bone quality have various advantages, including non-invasiveness, multiple measurements. Especially, the development of advanced imaging techniques allows the assessment of bone quality at the three-dimensional (3D) microstructure level, such as QCT, micro-CT, high-resolution magnetic resonance imaging (HR-MRI).

### X-ray-based modalities

#### Radiography

Traditional radiography is a cost-effective, widely available method for examining bone geometry, structure, and fracture risk that has been utilized in a wide range of studies [10–16]. The fracture toughness of bone tissue is highly connected to the bone shape defined by parameters based on plane X-ray radiogrammetry [11]. Furthermore, cortical thickness (CTI) and cortical-medullar index (CMI), as predictors of bone quality, can be obtained from anteroposterior radiographs [10, 15]. Tingart et al. [15] used anteroposterior radiographs to measure the CTI of 19 human cadaver humeri. The results indicated that the CTI of the proximal diaphysis can be a reliable indicator of the bone quality of the proximal humerus. Moreover, the CTI measured by radiographs has a significant positive correlation with BMD evaluated by DEXA. Clavert et al. [10] tested the CMI of 21 cadaveric distal humeri by plain radiographs showed that it is a predictor of the bone quality of the distal humerus and has a significant positive correlation with BMD measured by DEXA and CT-scan (pQCT). Aside from CTI and CMI, the trabecular homogeneity index (THI) was also used to assess bone quality using a plain radiograph, and it shows a strong connection with DEXA and CT-derived data [12]. However, radiography has its limitations, such as low sensitivity, unable to further visualize the microstructure of bone specimens, and that only 2D images are available.

#### Dual-energy X-ray absorptiometry (DEXA)

DEXA can provide an integrated examination of cortical and trabecular bone, which is frequently practiced in routine practice [10, 17–21]. Choel et al. [17] used 63 mandibular bone specimens to investigate the potential utilization of DEXA for the assessment of bone mineral content (BMC) and BMD prior to implant placement. Furthermore, Hua et al. [18] used 19 mandibular bone samples to evaluate the accuracy of fractal analysis and morphometry measured by DEXA. However, the limitations of the DEXA technique also need to be considered. Yang et al. [19] suggested that BMD measured by DEXA is only one aspect of the complex understanding of bone quality. Tan et al. [20] used 189 human lumbar specimens to verify and quantify the difference in DEXA-BMD between unexplained (in situ) and explanted (in vitro) scans. They found that the in vitro BMDs of the specimens were lower than those of in situ scans. This implied that several factors can affect the accuracy of the DEXA technique, such as the process of preparation, the surrounding soft tissue and their composition, and the scanning conditions. Additionally, Johannesdottir F [21] found that the aBMD of the femoral neck by DEXA (R^2^ = 0.69) was significantly lower than bone strength measured by QCT-based FEA in predicting femoral failure load.

### CT-based modalities

#### Quantitative computed tomography (QCT)

As a reliable and accurate technique, QCT and HR-pQCT scans have been used in the laboratory and provide us with valuable and comprehensive insight into bone quality [10, 22–32]. A study by Wachter et al. [30] concluded that QCT is a better predictor for the mechanical strength of the intertrochanteric region with objectivity and high precision. Liu et al. [28] reported that microstructural measurements and mechanical characteristics of the distal tibia can be efficiently derived from HR-pQCT images. Also, HR-pQCT is a promising tool for assessing the fracture healing process at the microscale [23].

Briefly, pQCT has emerged as an accurate technique for measuring bone quality with multiple advantages, including measured density-independent of overlying tissue, less susceptible to interference from bone size, relatively safe, higher accuracy, and 3D visualization [26, 31, 32]. Compared to the first-generation HR-pQCT with a nominal isotropic voxel size of 82 μm, the second-generation HR-pQCT has been improved to 61 μm, which allows a more accurate assessment of trabecular thickness (Tb.Th) [24].

#### Micro-computed tomography (Micro-CT, μCT)

Micro-CT is an advanced imaging modality for quantifying bone quality with high resolution. Currently, it has been gradually applied to assess the bone quality of human bone specimens, with a range of isotropic voxel resolution from 9 to 37 μm [14, 25, 28, 33–47]. Micro-CT imaging technique has higher accuracy compared to HR-pQCT, and it is considered as the “gold standard” in bone quality assessment, which allows objective and quantitative evaluation of trabecular bone structure [28, 38, 46]. Moreover, the combination of micro-CT images and mechanical tests can provide valuable and comprehensive information about the microarchitecture and microdamage of human cancellous bone specimens [41, 43]. To explore the influences of osteoporosis and gender on the microstructure of bone grafts, Xie et al. [35] used micro-CT to measure several important microstructure parameters, including bone volume fraction (BV/TV), bone surface density (BS/TV), specific bone surface (BS/BV), trabecular thickness (Tb.Th), trabecular number (Tb.N), trabecular separation (Tb.Sp), structure model index (SMI), and connectivity density (Conn.D.). This non-invasive technique may be sufficient to enhance the evaluation of the bone quality of human bone tissue.

### MR-based modalities

The MR-based modalities are a promising tool for evaluating bone morphometry due to their non-invasiveness, and high innate contrast between bone and soft tissue [46, 48–50]. Applications of MR-based scanning include nuclear magnetic resonance (NMR), high-resolution MRI (HR-MRI), and micro-MRI (μMRI).

Link et al. [49] compared parameters of the trabecular bone structure obtained from HR-MRI and multi-slice computed tomography (MSCT) with 39 distal radius bone specimens. Their data indicated that structure parameters derived from HR-MRI performed better in the prediction of trabecular bone structure, although this technology is more susceptible to image post-processing. In addition, the distribution and changes of water within bone tissue in relation to bone quality (i.e. bone strength and toughness) were also investigated by nuclear magnetic resonance (NMR) [48]. The findings demonstrated that quantification of mobile and bound water by MR imaging techniques could potentially serve as an indicator of bone quality. Correspondingly, Ni et al. [48] reported that the distribution of bound and free water measured by NMR could be considered as an important factor to determine bone quality. For microstructure analysis of micro-MRI images, Liu et al. [46] validated the 3D model-independent microstructure measurements by micro-MRI and micro-CT. They concluded that the microstructural and mechanical properties of most bone specimens could be efficiently derived from micro-MRI, as well as provide additional information on bone quality.

### Mechanical testing methods

#### Traditional testing

In addition to indirect imaging modalities, traditional mechanical methods can provide an accurate and direct assessment of bone quality at the tissue level, such as structural stiffness, bone strength, elastic modulus, and ultimate stress [51–55]. Several studies quantitatively investigate the changes in microstructure and morphology of human bone specimens using a combination of mechanical testing methods and micro-CT [56–60].

A uniaxial compression test was employed by Kalouche et al. [55] to determine the mechanical characteristics of glenoid cancellous bone in the three planes (axial, coronal, and sagittal). Bayraktar et al. [51] compared the mechanical properties at the tissue level by compression and tensile tests using human trabecular and cortical bone specimens. Charlebois et al. [56] studied 148 bone specimens from different anatomical regions using unconfined and confined compression methods. The data on the behaviour of human trabecular bone at large strain under compression indicated that the influence of tissue fabric would decrease with strain and plays a significant role in the softening behaviour of bone tissue. Furthermore, the application of mechanical loading also allows the microdamage of bone specimens to be studied, which is an aspect of bone quality [59, 60]. Lambers et al. [57] suggested that microdamage has a greater impact on the bone quality of human cancellous bone. Hence, the application of these traditional testing methods can provide more direct data on bone quality at both the tissue and micro-levels when combined with micro-CT.

#### Indentation testing

Currently, micro-indentation testing can measure bone properties at the millimeter level, and nano-indentation testing has the potential to measure the mechanical properties of bone at the level of trabeculae or osteons. These novel techniques are being used in vitro to evaluate bone quality at various anatomical sites [5, 6, 61–67].

A study by Dall'Ara et al. [5] concluded that micro-indentation has the ability to distinguish between severely damaged and intact tissue for human vertebral bone tissue. Jenkins et al. [63] claimed that reference point micro-indentation (RPI) can be used as a useful tool for evaluating the mechanical properties of bone in the laboratory. Two studies directly compared RPI with traditional mechanical tests (bending test) [66, 67]. Granke et al. [66] claimed that RPI properties are likely to be influenced by both elastic and plastic behaviour of bone tissue. However, Krege et al. [67] reported that the RPI technique alone is not sufficient to evaluate the mechanical properties of bone.

For nanoindentation, it provides a novel perspective that has been applied to the research on bone materials, especially for volumes as small as lamellae [6, 65]. Albert et al. [6] used nanoindentation to investigate the effects of disease severity (osteogenesis imperfecta) on the local elastic modulus and hardness of bone tissue. The nanoindentation technique makes it possible to investigate the characterization of bone material properties and evaluate modulus and hardness at a smaller scale.

### Compositional characterization

As mentioned above, water within bone tissue has a certain effect on bone quality (i.e. bone strength and toughness), which can be measured by NMR. But additionally, compositional characterization (i.e. DBM and organic compositions) is generally acknowledged as being important.

#### Degree of bone mineralization

The mineralization process consists of a primary deposition of mineral substance on the calcification front, followed by a slow and progressive increase in mineral deposition named secondary mineralization [68]. According to Follet et al. [68], the more mineralized the cancellous bone, the greater the stiffness and compressive strength. Although the increase in DBM may make bone stiffer and more resistant to mechanical loading, too high a mineral to matrix ratio would result in increased brittleness (i.e. higher tendency to crack propagation), and decreased toughness (i.e. the ability to deform without fracturing). Contrastly, this ratio that is too low can lead to bone softening, reduced stiffness and strength. Saito et al. [69] reported that DBM is related to distinct patterns of enzymatic and non-enzymatic cross-links in human bones and is an important element in assessing bone quality.

#### Organic composition

The ability of bone strength is not only determined by DBM, but also by organic composition (i.e. collagen glycation, collagen cross-links), which has been explored in various studies [47, 70–75]. As the major organic interagent, type I collagen is vulnerable to enzymatic and non-enzymatic biomechanical alterations that impact bone quality in numerous ways [47, 73]. The testing data by Poundarik et al. [72] indicated that advanced glycation end products (advanced glycation end products, AGEs) created by non-enzymatic glycation could be used for diagnostic applications in fracture risk assessment. Ural et al. [70] measured total fluorescent AGEs from 96 human cortical bone specimens indicated that AEGs may contribute to bone fragility by altering bone matrix properties. Furthermore, the extent of non-enzymatic glycation (NEG) is linked to alterations in the microarchitecture and microdamage of cancellous bone [73]. Willett et al. [71] used hydrothermal isometric tension (HIT) to measure the collagen’s thermal stability and network connectivity in order to observe the correlation between bone collagen integrity and fracture toughness of cortical bone. They found that the integrity of bone collagen is a critical factor for the fracture toughness of cortical bone. Therefore, the investigation of the bone matrix at the microstructure, and in particular collagen, plays a fundamental role in the mechanical properties of bone tissue at the macroscopic level.

## Discussion

The application of each method is closely related to the study design and the outcomes of interest. The X-ray-based imaging methods, including radiography and DEXA, have the advantages of low-cost, low-radiation. However, their limitations make it impossible to provide comprehensive and accurate information on bone quality. For CT-based techniques, including QCT, HR-pQCT, and micro-CT, they can perform 3D image reconstruction and microstructure analysis of human bone specimens, which enables more accurate and comprehensive bone quality information. Many studies have taken micro-CT imaging analysis as the “gold standard” of bone quality assessment [28, 38, 46]. The micro-CT technique can be considered as a comprehensive, high-resolution, three-dimensional, non-invasive technique for the assessment of bone microstructure. Moreover, the development of advanced MRI-based techniques, such as NMR, HR-MRI, micro-MRI, has shown promising results in the assessment of bone structure and water composition, providing additional information [46, 50]. However, most of the advanced imaging techniques described in this review are limited to a minority of laboratories due to expensive equipment and professional operation. Related research and technological breakthroughs need to be explored in order to make these novel imaging techniques to in vivo research and eventually to the clinic.

Compared to indirect imaging techniques, conventional mechanical methods can directly provide the performance of whole bone or bulk tissue specimens. Nevertheless, they are used only for ex vivo bone specimens due to the nature of destruction. However, with the development of indentation techniques, it is possible to directly test bone quality in a minimally invasive manner [5, 62, 66]. This technique has the advantages of being direct, simple, minimally invasive, as well as allowing in vivo testing. The shortcomings are that its results are relatively sole (only tissue hardness and brittleness) [66] and are limited to superficial sites, such as the tibial midshaft. Also, the reliability and significance of the parameters need to be validated further [76]. From our perspective, the combination of imaging modalities and mechanical testing methods would be a good choice for the assessment of bone quality ideally and comprehensively at both micro- and tissue levels.

Furthermore, compositional characterization, both DBM and organic composition, plays an essential role in assessing the mechanical properties of bone tissue and can provide more fundamental information that yields mechanistic insights into affecting bone quality [68, 69, 71]. Especially for bone collagen, there is a significant association with clinically relevant bone diseases, such as osteoporosis, osteogenesis imperfecta, and diabetes-related diseases [77, 78].

Previous studies have reported that collagen content in human bones reaches a maximum during adolescence and gradually decreases thereafter with aging [77, 79]. Compared with age-matched healthy subjects, osteoporotic bone indicated that reductions in the enzymatic cross-links and an increase in AGEs cross-links in bone [69, 80]. In diabetic bone tissue, BMD may be normal, but bone strength has decreased, which correlates with the increased formation of AGEs [77, 81]. For osteogenesis imperfecta, it is also a disease closely associated with collagen, and studies have shown that the orientation of collagen is highly disordered and that the collagen-mineral particle network is profoundly altered [78]. Therefore, the alteration of bone quality and biomechanical performances is the macroscopic result of a sequence of composition and microstructural events.

There are several limitations to our systematic review. First, not all test methods and studies were summarized in our review, which is a limitation of all systematic reviews. In this article, “bone quality” and “human bone specimens” were used as search terms. Actually, “bone quality” is not universally defined, and there are several other interchangeable phrases used, including “bone material quality”, “bone matrix quality”, etc. Similarly, the search term “human bone specimens” is interchangeable with “human bone samples”. Due to the limitation of content, it is difficult, or even almost impossible, to use all possible search terms in a review.

In order to further expand the search, the “similar articles” option of PubMed and the references for main articles were used in this review. Second, there is the risk of selection bias since the presence of heterogeneous. Finally, this review focuses only on imaging techniques, mechanical testing methods, and the effects of compositional characterization, computational techniques such as FEA are not included.

## Conclusions

Advanced techniques are playing an increasingly important role due to their multiple advantages, focusing on the assessment of bone morphology and microarchitecture. Non-invasive imaging modalities and mechanical testing techniques, as well as the assessment of bone composition, need to complement each other in order to provide comprehensive and ideal information on the bone quality of human bone specimens.

## Supplementary Information

**Additional file 1.** The adapted Newcastle-Ottawa Quality Assessment Scale was used for quality assessment of the included studies.

## Data Availability

All the data of the manuscript are presented in the paper or additional supporting files.

## Abbreviations

CTI: Cortical thickness
2D: Two-dimensional
3D: Three-dimensional
DEXA: Dual-energy X-ray absorptiometry
BMD: Bone mineral density
BMC: Bone mineral content
μCT: Micro-computed tomography
μMRI: Micro-magnetic resonance imaging
BV/TV: Bone volume fraction
Tb.Th: Trabecular thickness
Tb.Sp: Trabecular spacing
Tb.N: Trabecular number
BS/TV: Bone surface density
SMI: Structure model index
Conn.D.: Connectivity density
HR-MRI: High-resolution magnetic resonance imaging
RPI: Reference point indentation
HR-pQCT: High-resolution peripheral quantitative computed tomography
PRISMA: Preferred Reporting Items for Systematic Reviews and Meta-Analyses
WHO: World Health Organization
FEA: Finite element analysis
NOS: Newcastle–Ottawa Scale
CMI: Cortical-medullar index
THI: Trabecular homogeneity index
NMR: Nuclear magnetic resonance
MSCT: Multi-slice computed tomography
DBM: Degree of bone mineralization
AGEs: Advanced glycation end products
NEG: Non-enzymatic glycation
HIT: Hydrothermal isometric tension

## Declarations

### Patient and public involvement

No patient involved.
