# Supplementary material for: Methods for bone quality assessment in human bone tissue: a systematic review
Source: J Orthop Surg Res. 2022 Mar 21;17:174. doi: 10.1186/s13018-022-03041-4 (PMC8935787; doi:10.1186/s13018-022-03041-4)
Supplement: Supplementary file 1 — Additional file 1. The adapted Newcastle-Ottawa Quality Assessment Scale was used for quality assessment of the included studies. [file 13018_2022_3041_MOESM1_ESM.docx]

**Selection** (Maximum 3 points)

1. Representativeness of anatomical sites or factors

a) anatomical site of the specimens reported. ★

b) no detailed description on anatomical site of the specimens.

c) no description.

2. Representativeness of parametric data

a) enough quantitative information to discriminate bone characteristics. ★

b) fairly information to discriminate bone characteristics

c) qualitative descriptive information.

3. Sample size:

a) Justified, including sample size calculation. ★

b) Not justified.

c) No information provided.

**Comparability:** (Maximum 2 points)

1. Comparability of test/controls on the basis of the analysis

a) for studies evaluating bone quality by different methods: clear comparison between cohorts or groups. ★

b) for studies evaluating bone quality: clear statement on bone specimen parameters. ★

c) no clear group assignments or clear statement.

**Outcome** (Maximum 6 points)

**1. Assessment of outcome**

a) independent blind assessment ★

b) record linkage

c) self-report

d) no description

**2. Assessment method**

a) Adequate analysis to show the outcomes ★

b) Adequate but not sufficiently validated

c) Inadequate

**3. Outcome description**

a) application of correlation among studied variables. ★

b) only descriptive data.

**4. Specimen information (age)**

a) the age information of the specimen was described in detail ★

b) not described or incomplete.

**5. Number of specimens**

a) the number of specimens was enough (more than the average, 42). ★

b) the number of specimens was limited (less than the average, 42).

**6. Statistical test:**

a) The statistical test used to analyze the data is clearly described and appropriate, and the measurement of the association is presented (including SD/SE and the probability level; p value). ★

b) The statistical test is not appropriate, not described or incomplete.
